# Differential Contributions of Intrinsic and Extrinsic Pathways to Thrombin Generation in Adult, Maternal and Cord Plasma Samples

**DOI:** 10.1371/journal.pone.0154127

**Published:** 2016-05-19

**Authors:** Nicklaus T. Rice, Fania Szlam, Jeffrey D. Varner, Peter S. Bernstein, Arthur D. Szlam, Kenichi A. Tanaka

**Affiliations:** 1 Department of Obstetric and Gynecology, Vanderbilt Medical Center, Nashville, Tennessee, United States of America; 2 Department of Anesthesiology, Emory University School of Medicine, Atlanta, Georgia, United States of America; 3 School of Chemical Engineering, Purdue University, West Lafayette, Indiana, United States of America; 4 Department of Clinical Obstetric & Gynecology and Women’s Health, Albert Einstein College of Medicine/Montefiore Medical Center, Bronx, New York, United States of America; 5 Department of Mathematics, CCNY, New York, United States of America; 6 Department of Anesthesiology, University of Maryland School of Medicine, Baltimore, Maryland, United States of America; Xavier Bichat Medical School, INSERM-CNRS - Université Paris Diderot, FRANCE

## Abstract

**Background:**

Thrombin generation (TG) is a pivotal process in achieving hemostasis. Coagulation profiles during pregnancy and early neonatal period are different from that of normal (non-pregnant) adults. In this *ex vivo* study, the differences in TG in maternal and cord plasma relative to normal adult plasma were studied.

**Methods:**

Twenty consented pregnant women and ten consented healthy adults were included in the study. Maternal and cord blood samples were collected at the time of delivery. Platelet-poor plasma was isolated for the measurement of TG. In some samples, anti-FIXa aptamer, RB006, or a TFPI inhibitor, BAX499 were added to elucidate the contribution of intrinsic and extrinsic pathway to TG. Additionally, procoagulant and inhibitor levels were measured in maternal and cord plasma, and these values were used to mathematically simulate TG.

**Results:**

Peak TG was increased in maternal plasma (393.6±57.9 nM) compared to adult and cord samples (323.2±38.9 nM and 209.9±29.5 nM, respectively). Inhibitory effects of RB006 on TG were less robust in maternal or cord plasma (52% *vs*. 12% respectively) than in adult plasma (81%). Likewise the effectiveness of BAX499 as represented by the increase in peak TG was much greater in adult (21%) than in maternal (10%) or cord plasma (12%). Further, BAX499 was more effective in reversing RB006 in adult plasma than in maternal or cord plasma. *Ex vivo* data were reproducible with the results of the mathematical simulation of TG.

**Conclusion:**

Normal parturient plasma shows a large intrinsic pathway reserve for TG compared to adult and cord plasma, while TG in cord plasma is sustained by extrinsic pathway, and low levels of TFPI and AT.

## Introduction

Procoagulant tendency in coagulation system during pregnancy and puerperium may be considered as a survival advantage, preventing hemorrhage after the delivery [1, 2]. However, it is well recognized that plasma hypercoagulability can be associated with the risk of miscarriage, and venous thromboembolism [3, 4]. Coagulation system in the fetus evolves according to the gestation time [5] and the function of coagulation (procoagulant and anticoagulant activities) plays key roles in the fetal development [6]. Under normal conditions, tissue factor (TF) is thought to be the initial trigger for the initiation of coagulation at the site of vascular injury. During the initial activation of (extrinsic pathway) coagulation, traces of activated factor X (FXa) and thrombin are generated, but they are susceptible to coagulation inhibitors such as tissue factor pathway inhibitor (TFPI) and antithrombin (AT) [7, 8]. Thus, the propagation of coagulation via thrombin-mediated feedback (intrinsic pathway) is only feasible at the local milieu where procoagulant responses overcome anticoagulant forces [9]. Physiological changes in coagulation during pregnancy, and neonatal coagulation system seem to render functionally different controls over coagulation compared to normal adults [5, 10–13]. However, there is a paucity of data on the regulation of thrombin generation (TG) in pregnant women, and neonates. We hypothesized that comparative evaluations of TG using an inhibitor of FIXa or TFPI would delineate the specific contribution of extrinsic and intrinsic pathways. Therefore, we measured plasma coagulation factors and inhibitors as well as endogenous TG patterns in the plasma obtained from parturients and neonates at term. Subsequently, we utilized the mathematical simulation of TG to model differences in adult, maternal and cord plasma.

## Methods and Materials

This study was approved by the institutional review board of the Montefiore Medical Center, and all subjects participating in this study gave informed and written consents prior to participation. Blood samples were obtained from 20 term pregnant women (maternal group). Immediately following delivery, ten ml of maternal or cord blood (cord group) samples were collected in 4.5 ml Vacutainer tubes (Beckton-Dickinson, Franklin Lakes, NJ) containing 3.2% sodium citrate. Additionally, blood samples were also collected from 10 healthy volunteers (adult group) after giving informed and written consents according to the protocol approved by Emory University Institutional Review Board. All volunteers denied any coagulation abnormalities, and none were receiving any medications that could affect coagulation testing. All blood samples were immediately centrifuged at 2000 x g for 20 min to obtain platelet-poor plasma, which was stored at -80°C until the batch analysis. Because of volume constraints 10 cord and maternal plasma samples were used for thrombin generation, and another 10 sets were used for coagulation studies. In adult, maternal and cord plasma samples, prothrombin time and activated partial thromboplastin time (aPTT) were performed. Additionally, the following coagulation factors (F) and inhibitors were quantified in maternal and cord plasma: prothrombin (FII), FV, FVII, FVIII, FIX, FX, FXI, protein C, and antithrombin (AT). All measurements were performed per manufacturer’s instructions using Diagnostica Stago kits and reagents designed for Stago Compact analyzer (all from Diagnostica Stago, Parsippany, NJ). Results of PT and aPTT testing were expressed in seconds (sec), and of coagulation proteins in % activity.

Anti-FIXa aptamer (RB006) was a kind gift from Regado Biosciences (Triangle Park, NC). It was supplied as 21mg/ml solution. RB006 is an RNA based aptamer conjugated to 40-kDA polyethylene glycol carrier to increase its half-life. For TG experiments, it was freshly diluted with normal saline (0.9%), and was used at the final plasma concentration of 24 μg/ml. BAX499 was a kind gift from Baxter Healthcare Corp. (Cambridge, MA), and it was also freshly prepared in normal saline, and was used at the final concentration of 200 nM. The addition of both drugs to plasma samples resulted in less than 1% dilution.

### Thrombin Generation in Plasma Samples

The calibrated automated TG assay (Thrombinoscope^TM^, Diagnostica Stago, Parsippany, NJ) was used to measure the rate and the amount of TG in plasma according to the change in fluorescence produced by the hydrolysis of a fluorogenic peptide (Z-Gly-Gly-Arg-AMC) by thrombin [14]. Briefly, to each well of a 96 well microtiter plate (Microfluor black, ThermoLabsystems, Franklin, MA), we added 80 μl of various PPP samples. Calibrator wells, in which 20 μl of thrombin calibrator was added to 80 μl of plasma sample, were run in parallel for each plasma. TG was triggered with 20 μl of 2 pM tissue factor (TF, final concentration in the well)-based PPP reagent, which was prepared by mixing of appropriate volumes of 1 pM and 5pM PPP reagents (Diagnostica Stago, Parsippany, NJ). TF at 2 pM was chosen for the experiments based on previous work with RB006[15]. The reaction was started by adding 20 μl/well of CaCl_2_–substrate buffer and was continuously monitored for 90 min. At the completion of measurements the acquired data was automatically processed by the system’s software (Thrombinoscope BV, Maastricht, the Netherlands), and then exported into the EXCEL software for further data processing and graphical display. The following TG parameters were investigated; Lagtime = Time required for initial thrombin generation (min), Cmax = peak thrombin (nM), and Rate = mean velocity index of thrombin generation (nM/min).

Based on previous work of Tanaka, *et al*. [16], RB006, an anti-FIXa aptamer (final concentration, 24 μg/ml), was also added to specifically inhibit FIXa (intrinsic pathway), and to induce hypocoaguable state similar to hemophilia B [15]. This concentration of RB006 has been shown to increase aPTT values by 2.4 to 2.8 fold when added to normal adult plasma [16], and this magnitude of aPTT prolongations by RB006 has been correlated with >90% loss in FIX activity in an *in vivo* study involving healthy normal volunteers [17]. In addition to native and RB006 supplemented plasma testing, BAX499 was used to evaluate the impact of down-regulating TFPI (extrinsic pathway inhibitor) on TG in plasma. Based on the work of Waters, *et al*., and Gorczyca, *et al*., [18, 19] we choose to use 200 nM of BAX499. This concentration has been shown to significantly attenuate the ability of TFPI to inhibit FXa activation by TF/FVIIa in normal adult plasma. This intervention was expected to enhance TF-mediated procoagulant responses. BAX499 effects on TG in plasma samples pre-treated with RB006 were also evaluated.

### Mathematical Simulation of Thrombin Generation

The mathematical model of TG was employed to further delineate the differences among TG patterns in adult, maternal, and cord plasma samples. The enzymatic reactions considered in the mathematical model were compiled from the previously published literature [20, 21]. The model of TG presented here (see Appendix for the description of the model and the equations) follows the reaction triggered by tissue factor (TF; thromboplastin). A rapid binding of activated FVII (FVIIa) to TF catalyzes initial FXa formation. The feedback activation of FV and FVIII by thrombin (0.1–1 nM) is considered to be the key procoagulant reaction (intrinsic pathway) to sustain TG on phospholipids (activated platelets). For mathematical simulation of coagulation, measured levels of plasma coagulation factors and inhibitors in maternal and cord plasma were used for comparison with normal adult plasma. For any factor or inhibitor that was not measured in our study, empirical data were used according to the literature [5, 11, 22, 23]. Time-dependent concentrations for TG using 2 pM of TF as a trigger were modeled in normal adult, maternal, and cord plasma. The effects of RB006 were simulated by attenuating the concentration of FIX (90% of adult FIX level inhibited), since RB006 binds with the same affinity to FIX as to FIXa, and by attenuating TFPI level to simulate BAX499 effect [18, 24]. The concentrations of FIX and TFPI were also modified from 0.1 to 200% while keeping the coagulation factors and inhibitors at the native levels for each plasma group, in order to simulate the corresponding changes in TG. Further, the differential effects of AT alone or in combination with TFPI in cord plasma were also evaluated.

## Statistics

All data are shown as mean±SD (standard deviation). Differences in coagulation factor and inhibitor levels between maternal and cord plasma were compared using independent t—test statistics. The effects of RB006 and BAX499 on thrombin generation in adult, maternal and cord plasma were compared using one-way Anova with Bonferroni correction. A P value of <0.05 was considered significant. All analyses were performed using Graph-Pad Prism, Version 5.0 (Graph-Pad software, Inc., San Diego, CA, USA)

## Results

### Demographic Information

Twenty term pregnant women were enrolled in the study. The average age of the mother was 30±6 years (range 20–39) with an average weight of 91±26 kg (range 59–159). No pregnancy related major complications were reported in any of the women, but two mothers had sickle cell trait, and another two had pregnancy-induced diabetes mellitus. The average hematocrit was 33.4±4.3%, and the average platelet count was 217±56 x 10^3^/μl (range 153–368 x 10^3^/μl). None of the mothers were receiving any antiplatelet or anticoagulant agents prior, or during pregnancy. All the pregnancies were full-term (39±1.3 weeks; range 37–41), and resulted in deliveries of healthy, normal babies (mean weight of 3503±430 g) with a median Apgar score of 9 at 5 min.

### Coagulation Factor and Inhibitor Levels

**Fig 1:**
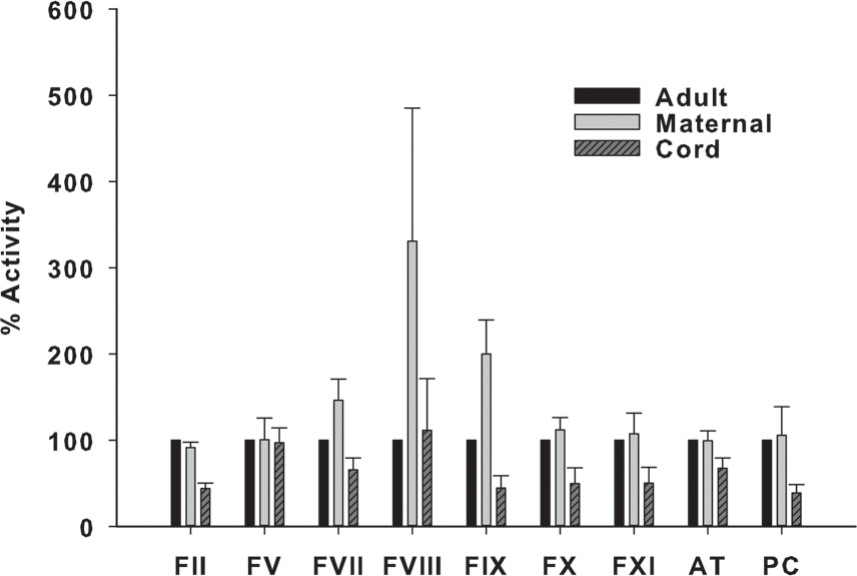
Maternal and cord coagulation factors and inhibitors. Adult levels set at 100% are shown for comparison. Maternal and cord values expressed as mean ± standard deviation (SD).

The measured coagulation factor and inhibitor levels in maternal and cord plasma samples are shown in % activity (**Fig 1**), and in nanomolar concentrations (**Table 1, S1 Table**). The adult levels were set to 100% activity (**Fig 1**). The levels of FVIII and FIX were respectively 3-fold and 2-fold higher in maternal than in adult plasma. Conversely, the average FIX level in cord samples was about one-fifth of maternal plasma level (44.4±14.3% in cord *vs*. 200±39.5% in maternal plasma). Further, the levels of prothrombin (FII), FIX, FX, and FXI were about 50% lower in the cord plasma than in adult plasma. FV and FVIII activities in cord plasma were in the normal adult ranges (97.7± 17.1%, 111± 60%, respectively). The cord plasma AT activity was below 70% of normal (1758 *vs*. 2600 nM), which was significantly lower (*P*<0.001 cord vs. maternal) than in normal adult and maternal plasma. Similar to other vitamin K-dependent factors, protein C level in the cord plasma was about 40% (*P*<0.001 cord vs. maternal) of normal adult and maternal plasma levels. PT values were prolonged in cord plasma, and aPTT values were shortened in maternal plasma (**Table 1**). On the other hand maternal PT values and cord aPTT values were still within normal ranges for healthy adults (10.8–13.5 sec, and 28.0–40.0 sec, respectively).

**Table 1:** Coagulation factor and inhibitor levels.

	PT (sec)	APTT (sec)	FII (nM)	FV (nM)	FVII (nM)	FVIII (nM)	FIX (nM)	FX (nM)	FXI (nM)	AT (nM)	PC (nM)
**Adult**	12.3±0.8	32.0±4.5	1400	20.0	0.20	0.30	70.0	135.0	30.0	2600	65.0
**Maternal**	13.2±0.6	25.2±2.6	1280	20.0	0.29	0.99	140.0	150.0	32.2	2590	68.6
**Cord**	15.6±1.3	35.9±5.3	616	19.5	0.13	0.33	31.0	67.0	15.2	1758	25.2

PT = prothrombin time, aPTT = activated partial thromboplastin time. Normal ranges: PT 11.0–14.0 sec, and aPTT 28.0–40.0 sec. PT and PTT values are shown as mean ± standard deviation. Factor (F) and inhibitor levels are expressed in nM.

### Thrombin Generation in Plasma

**Fig 2:**
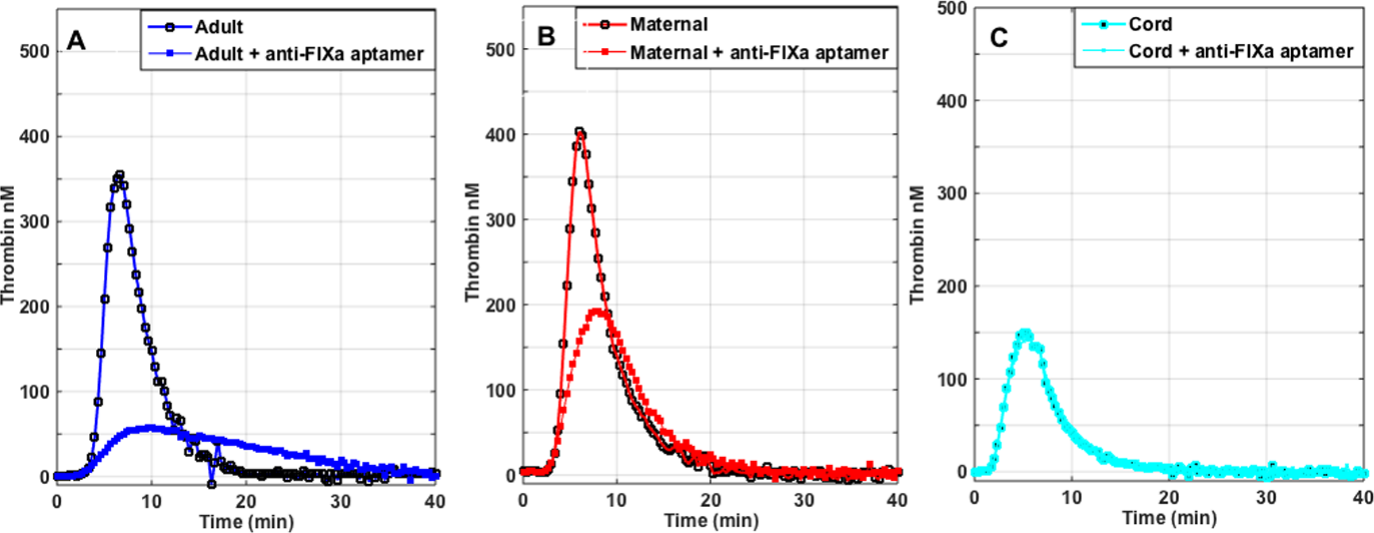
**Panels A-C.** Representative graphs of the effects of 24 μg/ml RB006 on thrombin generation in adult (A), maternal (B) and cord plasma (C). RB006 = anti-FIXa aptamer, 24 μg/ml.

**Fig 3:**
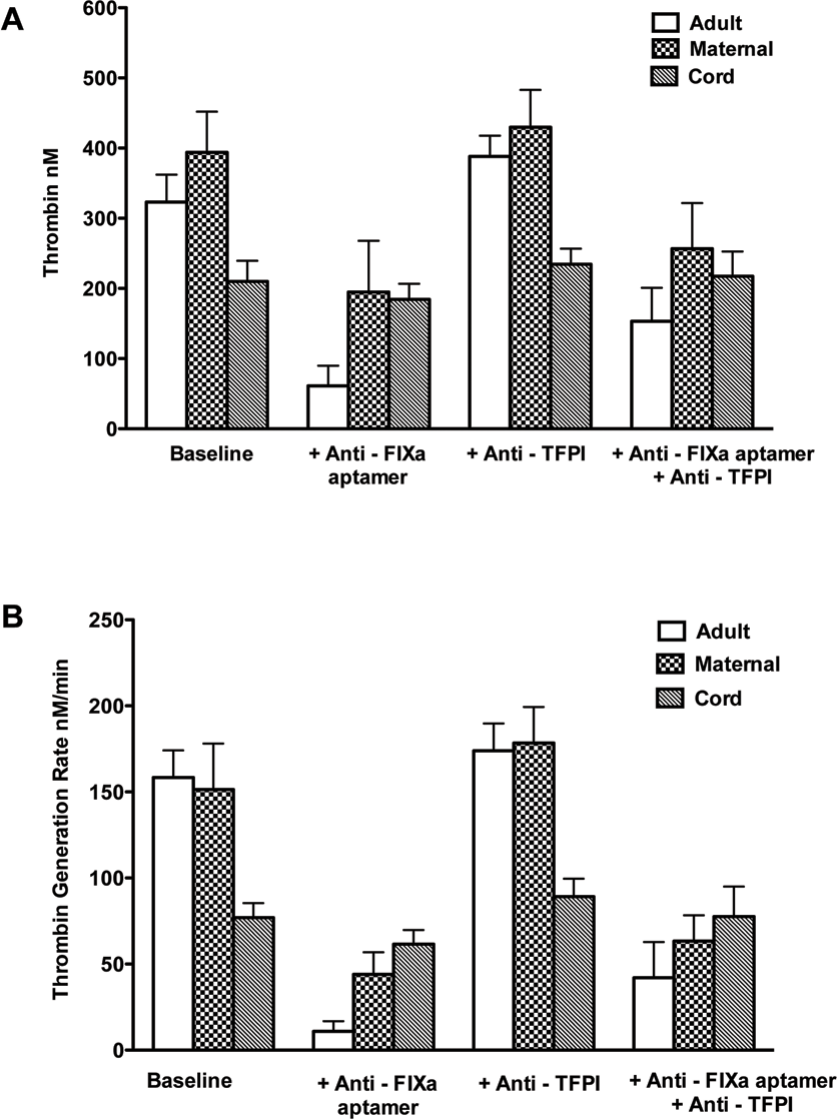
**Panels A-B.** Average thrombin generation (A) and rate (B) in adult, maternal and cord plasma. Anti-FIXa aptamer = RB006, 24 μg/ml; Anti-TFPI = BAX499, 200 nM.

The results of TG triggered by 2 pM of TF in adult, maternal, and cord plasma are summarized in **Table 2**. The average peak of TG in maternal samples was 22.0% higher than in normal adult, and was almost ~2-fold higher than in the cord plasma. Likewise the lagtime of TG was significantly shorter in cord plasma *vs*. maternal (*P* = 0.001) or adult plasma (*P* = 0.003) (**Table 2**). The addition of the anti-FIXa aptamer (RB006, 24μg/ml) decreased peak TG by 80.5±10.1% in adult samples vs. 51.1±16.1% in maternal plasma, but only by 12.4±6.9% in cord plasma (**Table 2**, **Fig 2A–2C**, **Fig 3A**). Thus the impact of FIXa inhibition as measured by the decrease in peak TG was as follows: adult > maternal > cord plasma. Conversely, the addition of TFPI inhibitor (BAX499, 200 nM) to plasma lead to increase of the peak TG by 20.7±6.7% in adult plasma, but smaller increases in maternal or cord plasma (9.7±7.0% in maternal, and 12.4±6.9% in cord plasma) (**Table 2, Fig 3A**). Adding BAX499 (200 nM) to RB006-treated samples was most effective in increasing peak TG in adult plasma (~151% increase) while smaller improvements were observed in maternal plasma (~31% increase), and cord plasma (~18% increase) (**Table 2, Fig 3A**). Adding BAX499 to RB006-treated samples shortened the lagtime in adult plasma samples (**Table 2, Fig 3A**), but had no effect on the lagtime of maternal or cord sample. RB006 had a profound inhibitory effect on the rate of TG in adult plasma (93% inhibition) but much lesser effect was seen in maternal or cord plasma (71% vs. 20%, respectively). Conversely, RB006 induced-decreases in the rate of TG in adult and maternal samples were incompletely recovered by the addition of BAX499, while the rate was normalized in the cord plasma (**Table 2**, **Fig 3B**).

**Table 2:** Effects of anti-FIXa (RB006) and anti-TFPI (BAX499) alone or in combination on thrombin generation parameters.

Sample	Peak Thrombin (nM)	Lag time (min)	Rate (nM/min)
**Adult**	323.2 ± 38.9	3.8 ± 0.5	158.4 ± 15.8
+ anti-FIXa	61.1 ± 28.7^†^	3.3 ± 0.3^†^	10.9 ± 5.9^†^
+ anti-TFPI	388.0 ± 29.6^‡^	3.1 ± 0.1^‡^	173.9 ± 15.9^‡^
+ anti-TFPI + anti-FIXa	153.0 ± 47.8^§^	3.1 ± 0.3^§^	42.1 ± 20.7^§^
**Maternal**	393.6 ± 57.9^#^	3.4 ± 0.5	151.4 ± 26.7
+ anti-FIXa	194.9 ± 72.9^†^	3.1 ± 0.3	43.9 ± 12.9^†^
+ anti-TFPI	429.7 ± 53.2	3.1 ± 0.5	178.4 ± 21.0^‡^
+ anti-TFPI + anti-FIXa	256.6 ± 65.0^§^	3.2 ± 0.4	63.3 ± 15.0^§^
**Cord**	209.9 ± 29.5*^¶^	1.9 ± 0.3*^¶^	77.0 ± 8.4*^¶^
+ anti-FIXa	184.3 ± 22.3^†^	1.8 ± 0.3	61.6 ± 8.2^†^
+ anti-TFPI	234.3 ± 22.2^‡^	1.7 ± 0.2	89.2 ± 10.4
+ anti-TFPI + anti-FIXa	217.4 ± 35.1	2.0 ± 0.4	77.6 ± 17.4

Data expressed as mean ± standard deviation. Adult versus maternal versus cord parameters compared using one-way ANOVA with multiple comparisons (Bonferroni t-test) with significance at *P*<0.05.

^#^Adult versus maternal plasma.

*Adult versus cord plasma.

^**¶**^Maternal versus cord plasma. Effects of the treatment within the group compared using repeated measures one way ANOVA with multiple comparisons (Bonferroni t-test) *vs*. pertinent comparator (adult, maternal or cord) with a significance at *P*<0.05.

^**†**^*vs*. anti-FIXa (RB006).

^**‡**^*vs*. anti-TFPI (BAX499).

^**§**^*vs*. anti-TFPI + anti-FIXa.

### Simulations of Thrombin Generation

**Fig 4:**
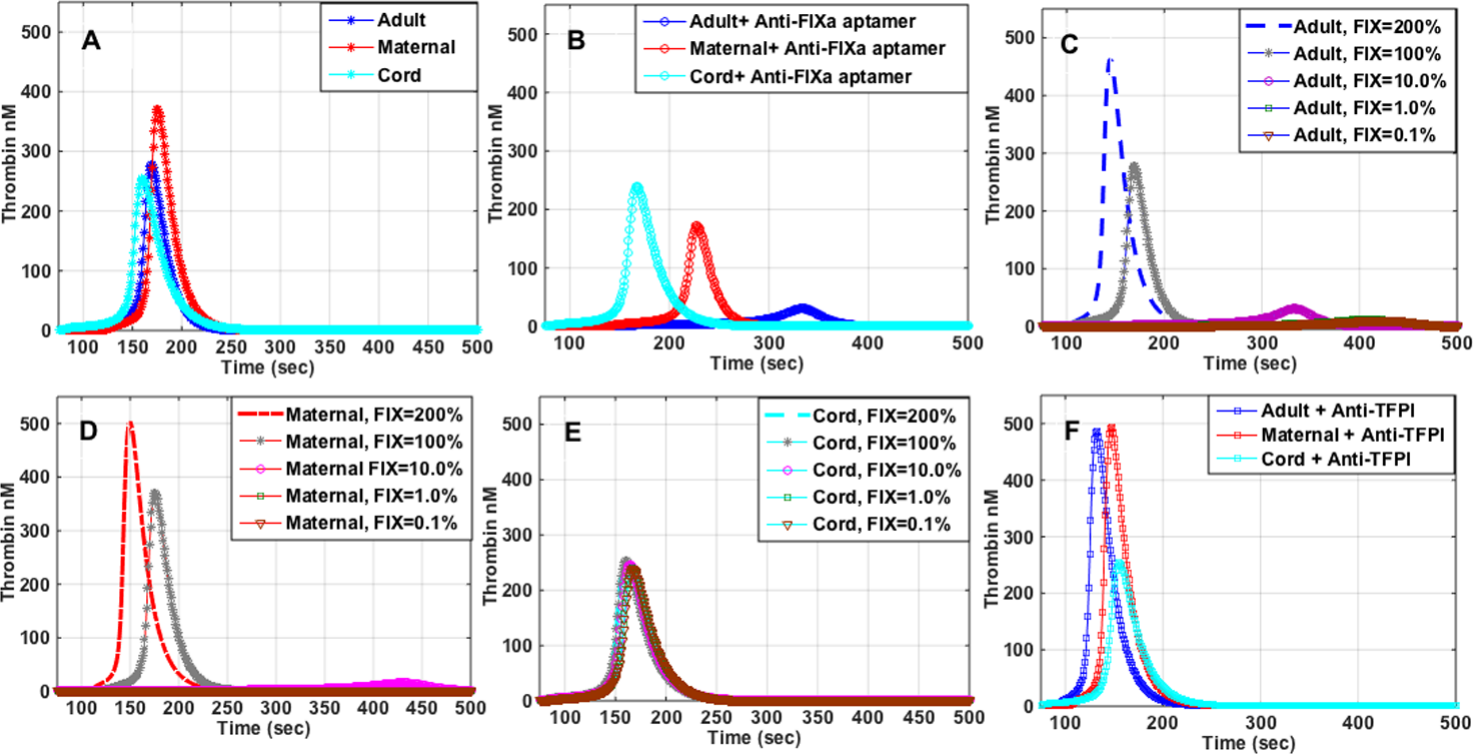
**Panels A-F.** Mathematical modeling of thrombin generation in adult, maternal and cord plasma. (A) native samples; (B) with anti-FIXa (RB006), 24 μg/ml; (C) with anti-TFPI (BAX499), 200 nM; (D-F) with varying levels (200%-0.1%) of FIX. 100% FIX activity equals 70 nM, 140 nM and 31 nM in adult, maternal and cord plasma, respectively.

**Fig 5:**
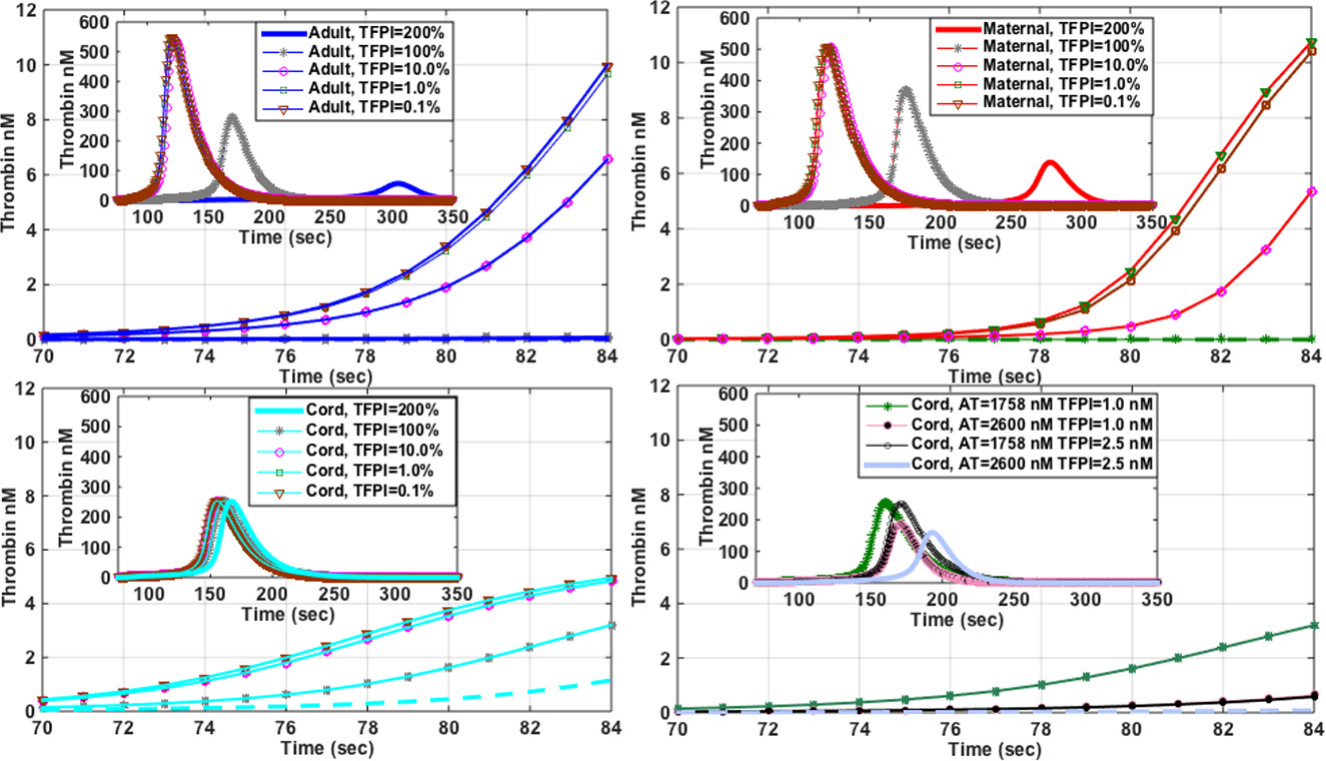
**Panels A-D.** Matematical modeling of differential effects of tissue factor pathway inhibitor (TFPI) on thrombin generation in the first 84 seconds in adult (A), maternal (B) and cord plasma (C). Effects of AT are also shown (D). Insets A-D = complete profiles of thrombin generation.

The peak of TG in maternal plasma was 1.3 to 1.5-fold higher than in adult or cord plasma, respectively (**Fig 4A**), but the lagtime was shortest in cord plasma. Further, concurrent with the *ex vivo* data, the simulated TG also demonstrated that the peak thrombin level was preserved (253 nM) even though the factor levels were significantly decreased in the cord plasma, particularly prothrombin (FII) (**Table 1**). This is due to lower levels of coagulation inhibitors as exemplified by AT (2600 nM in adult plasma compared to 1758 nM in the cord plasma, **Table 1**). As in the *ex vivo* data cord plasma TG appeared to be insensitive to lower FIX levels compared to adult and maternal plasma (peak TG decreased by 6% *vs*. 89% in adult, and 54% in maternal plasma) (**Fig 4B–4E**). Simulating BAX499 TFPI effect (decrease in TFPI) in the adult plasma resulted in an increase in the peak TG from 280 nM to 490 nM in the adult plasma, a 75% increase (**Fig 4F**). On the other hand, this effect was much smaller in the maternal plasma (~32%), and minimal in the cord plasma (**Fig 4F**). Additionally, the computer-simulated TG revealed differential effects of the TFPI and AT on TG generation patterns in adult, maternal and cord plasma. The lagtime of TG was shortest and the onset of thrombin peak was more rapid in cord plasma (**Fig 5A–5C**). However, the overall TG was less, and not as sensitive to decreasing concentrations of TFPI compared to adult or maternal plasma. This sensitivity was very much potentiated as shown by a decreased thrombin peak and an increased lagtime, when both AT and TFPI were adjusted to adult levels (**Fig 5D**).

## Discussion

In the current study, we demonstrate the distinct contributions of the intrinsic and extrinsic (TF) pathway, to thrombin generation in adult, maternal and cord plasma by the targeted inhibition of FIXa (using RB006) and TFPI (using BAX499, formerly ARC19499). Both RB006 and BAX499 are novel aptamer-based coagulation modifiers, which are single stranded nucleic acid ligands that can fold into a complex 3-dimensional structure, and bind to target proteins with high affinity and specificity [25, 26]. RB006 inhibits intrinsic pathway coagulation by binding specifically to FIXa and blocking FVIIIa-FIXa catalyzed conversion of FX to FXa [25], whereas BAX499 promotes coagulation by inhibiting TFPI [18], a main regulator of TF-FVIIa/FXa complex (extrinsic pathway) [25, 27]. In our *ex vivo* experiments, peak TG levels were highest in the parturients’ plasma compared to the adult plasma, and the lowest peak TG was found in the cord plasma. The rate of TG was ~2-fold higher in maternal and adult plasma than in the cord plasma, but the lagtime was shortest in the cord plasma.

Other investigators have also shown faster, but diminished TG in cord plasma [23, 28, 29]. TG assay results are influenced not only by procoagulant factors, but also by the levels of coagulation inhibitors. For example, lower TFPI or AT activity increases peak TG levels [28, 30]. The peak of TG is also strongly affected by prothrombin levels [31], and the lower mean prothrombin level (44% of normal adult) in our cord plasma contributed to the lower peak TG.

On the other hand, a 3-fold increase in concentration of FVIII along with a moderate increase in FVII, and a 2-fold increase in FIX were observed in the maternal plasma (**Table 1**). These values are consistent with the previously published maternal coagulation data [10, 11, 32]. Elevated levels of FVIII are known to promote TG [33]. These changes in coagulation factor levels in maternal and cord plasma partly explain prolonged PT in cord plasma, and the shortened aPTT in maternal plasma (**Table 1**).

The addition of RB006 (24 μg/ml) to plasma was much more effective in reducing both the rate and peak of TG in adult and maternal plasma than in cord plasma. The lower efficacy of RB006 in decreasing TG in maternal plasma is not unexpected because of the increased level of FIX along with FVIII compared to adult sample. The elevated levels of procoagulant factors FVIII and FIX at the time of delivery have been previously reported [11, 34]. This finding also underscores the importance of intrinsic tenase (complex of FVIIIa and FIXa) in the maternal hemostasis after the delivery. On the other hand, the observed insensitivity of the cord plasma to anti-FIXa aptamer is somewhat surprising because FIX levels were less than 50% of adult level (**Table 1**). However, Fritsch, *et al*. previously evaluated TG patterns in FVIII-depleted neonatal plasma, and reported that they were only slightly decreased relative to normal neonatal plasma [28]. Taken together, these data implicate that the driving force of TG in the cord plasma is extrinsic pathway because it is less prone to the inhibition due to lower levels of TFPI and AT [5, 28]. The inhibition of TFPI by BAX499 (200 nM) caused approximately 21% increase in peak TG in adult plasma, but the increments were only about 10–12% in maternal and cord plasma. It is thus possible that TFPI inhibition reaches a plateau in affecting TG according to the circulating levels of TF, TFPI, AT, or residual intrinsic pathway factor activity. This speculation is partially supported by the results of combination of BAX499 (TFPI inhibition), and RB006 (FIXa inhibition). TG levels and rates of TG in normal adult plasma samples were most profoundly affected by FIXa inhibition, and were significantly recovered after adding BAX499 (**Table 2**). In maternal plasma with high circulating FVIII and FIX, TG levels and rates of TG were less affected by FIXa inhibition, and the recovery of both TG parameters (*i*.*e*., peak and rate) with BAX499 was less than in normal adult plasma. In cord plasma with lower TFPI activity, FIXa and TFPI inhibition had the least effects on TG.

To further evaluate the effects of FIXa and TFPI inhibition in adult, maternal and cord plasma, we employed a computational coagulation model [21] to which we inputted pro- and anti-coagulant factor levels determined in the respective plasma samples. The mathematical simulation of TG in adult, maternal and cord plasma demonstrated similar TG patterns to *ex vivo* data, showing that TG was highest in maternal plasma and fastest in cord plasma. Further, the results of TG simulation of inhibition of FIXa (low FIX) in adult, cord and maternal plasma agreed very well with the laboratory data. Based on recent work of Sullenger, *et al*. [24], RB006 binds with similar affinity to FIX and FIXa. Using the model, and simulating the effects of sequential decrease of FIX (from 200 to 0.1% of the respective values, *e*.*g*., from 140 to 0.07 nM in adult), showed that the cord plasma was indeed much resistant to the effects of very low levels of FIX (**Fig 4E**). In other words, there was a very little difference in peak TG levels in cord plasma at 200% and 0.1% of FIX activity (cord factor IX levels, 62 and 0.031 nM, respectively). On the other hand, simulating the procoagulant effects of TFPI antagonism in adult plasma showed a much greater effect on increasing peak TG levels in comparison to the *ex vivo* data, results which are much closer to the data reported by Waters, *et al*. [18]. When TFPI concentrations were decreased from 200% to 0.1% of the respective values for adult, maternal and cord plasma (**Fig 5A–5C**), simulated TG patterns demonstrated the importance and the effectiveness of the TFPI in the regulation of thrombin generation, and specifically its influence on the lagtime, and in the initial rate of TG.

There are several limitations in this study. First, coagulation assays used in our experiments are static, plasma-based assays and thus coagulation *in vivo*, which involves platelets, monocytes, blood flow and endothelial responses cannot be fully appreciated. Secondly, blood samples were obtained from apparently healthy adults and parturients, and thus our results cannot be extended to any pathological bleeding or thrombotic state.

In summary, TG patterns in maternal plasma are characterized by the large reserve of intrinsic pathway, and reduced sensitivity to TFPI compared to those in normal adult plasma (possibly due to higher TFPI) [22]. In cord plasma, TG is mainly driven by extrinsic pathway, and is sustained by lower levels of TFPI and AT. However, lower vitamin K dependent factors levels limit the overall TG, therefore neonates may be susceptible to bleeding in case of hemodilution [35]. Our present data could be used as a basis for future investigations of target-specific hemostasis and antithrombotic therapies in parturients and newborns.

## Appendix

The mass balance equations were written around each protein (*e*.*g*., fXa) or protein complex (*e*.*g*., fXa-fVa) yielding the set of differential equations (vector from):

$$\begin{array}{l} \frac{dx}{dt}=f=S\cdot r(x,k)\\ x(t_{0})=\;x_{0} \end{array}$$ (1)

The symbol **S** denotes the stoichiometric matrix (193 x 301), **x** denotes the concentration vector of proteins or protein complexes (193 x 1), **k** denotes the vector of kinetic parameters (301 x 1) and **r** (**x**, **k**) denotes the vector of reaction rates (301 x 1). Each row in **S** describes a particular protein or protein complex, while each column describes the stoichiometry associated with a specific interaction in the network. Thus, the (*i*,*j*) element of S, denoted by σ_*ij*_, describes how protein *i* is connected to rate process *j*. If σ_*ij*_ < 0, then protein *i* is consumed in r_*j*_; conversely, if σ_*ij*_ > 0, then *i* is produced by r_*j*_; and if σ_*ij*_ = 0, there is no connection between protein *i* and rate process *j*. We have assumed mass action kinetics for each interaction, and the rate expression for the general reaction *q*:

$$\sum_{j\in \{R_{q}\}}\sigma_{jq}X_{j\to }\;\sum_{k\in \{P_{q}\}}\sigma_{kq}X_{q}$$ (2)

is given by:

$$r_{q}\;\left(x,k_{q}\right)\;\to \sum_{j\in \{R_{q}\}}k_{q}\Pi x_{j}^{-\sigma jq}$$ (3)

where {***R***_*q*_} denotes the set of reactants for reaction *q*, {***P***_*q*_} denotes the product set for reaction *q*, k_*q*_ denotes the rate constant governing the *q*th reaction, and σ_*jq*_, σ_*kq*_ denote stoichiometric coefficients (elements of the matrix **S**). We have treated every rate as nonnegative; all reversible reactions in the data source were split into two irreversible reaction steps. Thus every element of the reaction rate vector **r**(**x**, **k**) takes the form shown in Eq 3.

The model equations were solved using the livermore solver for ordinary differential equations (LSODE) routine of the OCTAVE programming environment (http://www.octave.org; version 2.1.71) on an Apple Computer MacOSX (http://www.apple.com; v10.5.3).[21]

## Supporting Information

S1 Table Factor levels, PT and aPTT data for maternal and cord plasma samples. (XLSX)
